# Hidden in plain sight: the impact of human rhinovirus infection in adults

**DOI:** 10.1186/s12931-025-03178-w

**Published:** 2025-03-28

**Authors:** Tommaso Morelli, Anna Freeman, Karl J. Staples, Tom M. A. Wilkinson

**Affiliations:** 1 Clinical and Experimental Sciences, Faculty of Medicine, University of Southampton, Southampton, UK; 2 NIHR Southampton Biomedical Research Centre, University Hospital Southampton, Southampton, UK

**Keywords:** Rhinovirus, Respiratory virus, Adults, Pneumonia, Asthma, COPD, Primary care, Hospitalised patients

## Abstract

**Background:**

Human rhinovirus (HRV), a non-enveloped RNA virus, was first identified more than 70 years ago. It is highly infectious and easily transmitted through aerosols and direct contact. The advent of multiplex PCR has enhanced the detection of a diverse range of respiratory viruses, and HRV consistently ranks among the most prevalent respiratory pathogens globally. Circulation occurs throughout the year, with peak incidence in autumn and spring in temperate climates. Remarkably, during the SARS-CoV-2 pandemic, HRV transmission persisted, demonstrating its resistance to stringent public health measures aimed at curbing viral transmission.

**Main body:**

HRV is characterised by its extensive genetic diversity, comprising three species and more than 170 genotypes. This diversity and substantial number of concurrently circulating strains allows HRVs to frequently escape the adaptive immune system and poses formidable challenges for the development of effective vaccines and antiviral therapies. There is currently a lack of specific treatments. Historically, HRV has been associated with self-limiting upper respiratory infection. However, there is now extensive evidence highlighting its significant role in severe lower respiratory disease in adults, including exacerbations of chronic airway diseases, such as asthma and chronic obstructive pulmonary disease (COPD), as well as pneumonia. These severe manifestations can occur even in immunocompetent individuals, broadening the clinical impact of this ubiquitous virus. Consequently, the burden of rhinovirus infections extends across various healthcare settings, from primary care to general hospital wards and intensive care units. The impact of HRV in adults, in terms of morbidity and healthcare utilisation, rivals that of the other major respiratory viruses, including influenza and respiratory syncytial virus. Recognition of this substantial burden underscores the critical need for novel treatment strategies and effective management protocols to mitigate the impact of HRV infections on public health.

**Conclusion:**

This review examines the epidemiology, clinical manifestations, and risk factors associated with severe HRV infection in adults. By drawing on contemporary literature, we aim to provide a comprehensive overview of the virus’s significant health implications. Understanding the scope of this impact is essential for developing new, targeted interventions and improving patient outcomes in the face of this persistent and adaptable pathogen.

## Introduction

Since its initial identification by Winston Price in the 1950s, human rhinovirus (HRV) has become recognised as one of the most common agents responsible for respiratory infections [1]. However, there are no approved therapies for this pathogen. The significance of HRV infection in children is well established, with consistent associations found between infections during childhood and the development of wheezing, asthma, and severe lower respiratory complications [2]. However, despite evidence pointing to substantial morbidity in adults, the impact of HRV infection in this population remains largely underestimated. This review aims to shed light on this impact by detailing the epidemiology, transmission, and clinical manifestations of HRV. It aims to update clinicians on the role of HRV infection beyond childhood, inform ongoing research efforts, and underscore the urgent need for innovative therapeutic approaches.

## Background

### Taxonomy

**Fig. 1:**
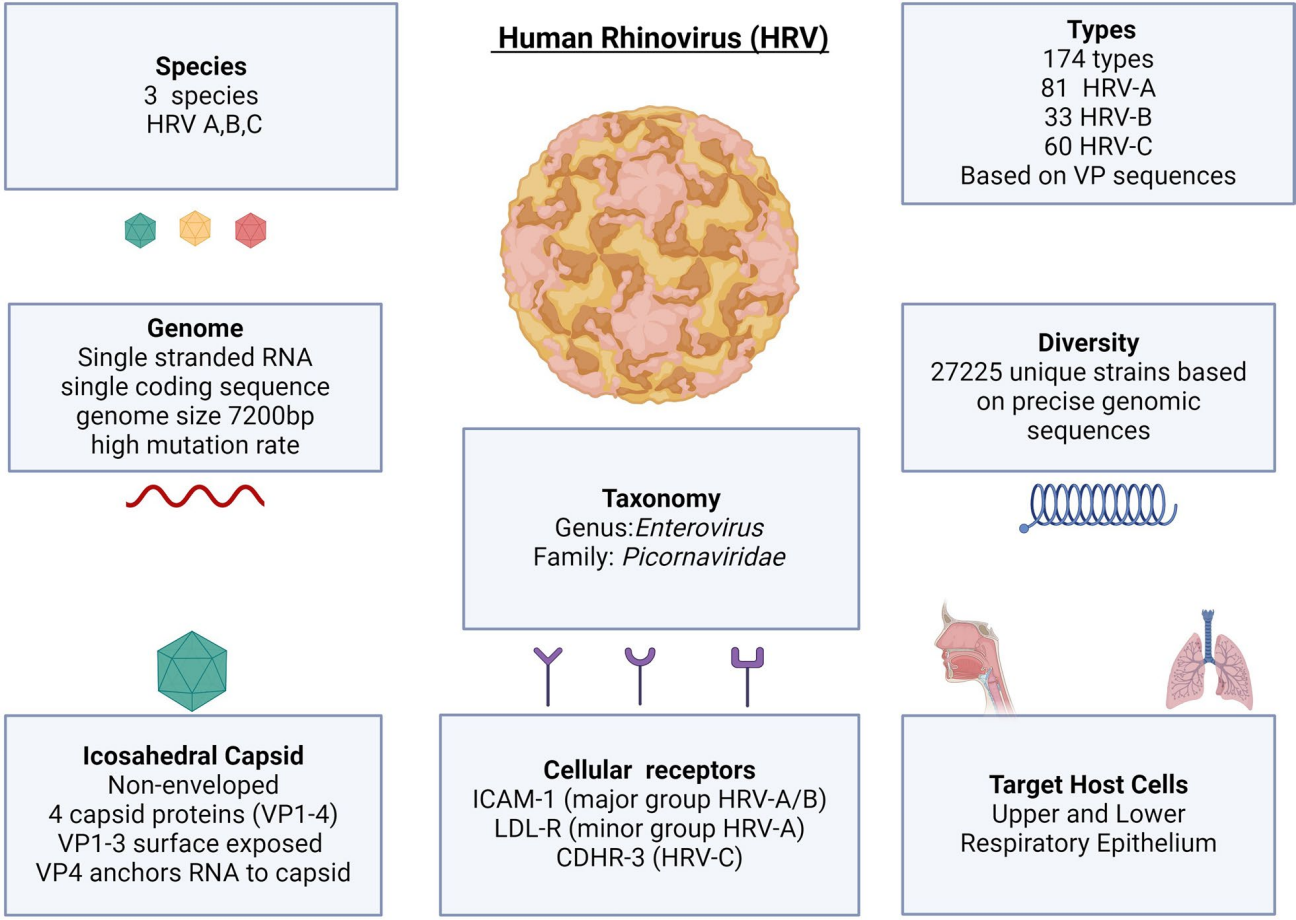
Overview of the structure, taxonomy, diversity, and infection targets of human rhinovirus (HRV). HRV is among the most diverse viral pathogens. The vast genetic diversity of circulating HRV strains hampers adaptive immunity, which is limited to homologous strains. Host defence is therefore largely dependent on innate responses. The co-circulation of these diverse strains also presents a challenge to the development of antiviral and vaccine therapies. Abbreviations: *bp* base-pairs, *CHDR-3* Cadherin Related Family Member 3, *ICAM-1* Intracellular Adhesion Molecule 1, *LDL-R* Low Density Lipoprotein Receptor, *RV* Rhinovirus, *RNA* Ribonucleic Acid, *VP* Viral Protein

HRVs are single-stranded, positive-sense RNA viruses belonging to the Enterovirus genus of the *Picornaviridae* family (Fig. 1).

HRVs comprise three primary species, namely, HRV-A, HRV-B, and HRV-C, each of which is distinguished by characteristic genomic features and phylogenetic sequences [3–5]. Notably, the discovery of HRV-C in 2006 was delayed due to its inability to be cultured using traditional techniques [6–8].

### HRV genome and diversity

The HRV genome is 7200 base pairs (bps) in length and consists of a single open reading frame flanked by a 5′ untranslated region (UTR) (~ 650 bp) and a 3′ UTR (~ 50 bp). The 5′ UTR is critical for replication and translation and contains essential structural and sequence elements, whereas the 3′ UTR contributes to transcriptional regulation. The translated protein is cleaved into 11 functional products, including four structural viral proteins (VPs), namely, VP1, VP2, VP3, and VP4, which form the capsid, and seven non-structural proteins involved in genome replication and assembly [5].

HRVs exhibit remarkable diversity, primarily due to their high mutation frequency during replication. Initially, HRVs were classified based on antigenic properties (serotyping), but the current classification relies on genotyping, utilizing divergence in nucleotide sequences of viral protein (VP) genes [9–12]. This genotyping approach has identified more than 170 HRV genotypes, which are further subdivided into more than 27,000 strains based on precise genomic sequencing differences [13].

In addition to frequent mutations, recombination also drives genetic diversity across *Picornaviridae*. However, HRVs exhibit lower recombination rates than non-HRV enteroviruses. Recombination in HRVs occurs predominantly in the 5′ untranslated region (UTR), which is essential for replication and translation, and occasionally in non-structural regions [14, 15]. Among the HRV species, HRV-A has the highest recombination frequency. HRV-C engages in sporadic interspecies recombination with HRV-A in the 5′ UTR, contributing to its genetic variability, whereas HRV-B exhibits minimal recombination activity, with few inter-genotypic exchanges documented [15, 16].

### HRV structure and replication

HRV is non-enveloped, with an icosahedral capsid composed of four proteins (VP1-4). VPs 1–3 form the external surface of the capsid, which possesses antigenic properties, while VP4 is located on the internal surface and is in direct contact with the viral genome [3, 17].

Intracellular adhesion molecule 1 (ICAM-1) is the viral receptor for all HRV-B types and most HRV-A types (known as the major HRV-A group) [3, 18]. Twelve types of HRV-A bind to low-density lipoprotein receptors (LDL-Rs), known as the minor HRV-A group [19]. Cadherin-related family member 3 (CHDR-3) acts as the glycoprotein viral receptor for HRV-C [3, 20].

Upon binding to their respective receptors, HRV enters airway epithelial cells via endocytosis and micropinocytosis. The more acidic intracellular pH triggers uncoating of the capsid, facilitating translation of the positive-sense viral RNA. Newly formed virions packaged with RNA are then assembled and subsequently released from the host cell to continue the infection cycle [21–23].

### Host immune response to HRV

Upon infection, pathogen-associated molecular patterns (PAMPs) present on HRV, such as elements of the HRV capsid and HRV-RNA, engage host pattern recognition receptors (PRRs), including Toll-like receptors (TLRs), retinoic acid inducible gene-1 (RIG-1) and melanoma differentiation associated gene 5 (MDA-5) [24]. RIG-1 and MDA-5 play crucial roles in host defence against RNA viruses by recognising HRV-RNA, leading to the induction of a type I/III interferon (IFN) response and the production of pro-inflammatory cytokines and chemokines. The subsequent recruitment of innate immune cells such as neutrophils, macrophages and dendritic cells precedes the slower mobilisation of the adaptive immune response. The balance between pro-inflammatory and anti-inflammatory signals determines the severity of the host response, ultimately influencing clinical outcomes. While HRV infection itself has limited direct cytopathic effects, IFNs, along with pro-inflammatory chemo/cytokines such as RANTES, ENA-78 IP-10, IL-6, and IL-8, are responsible for symptom development and cytotoxicity [24–30]. For instance, the level of IL-8 in nasal fluid is correlated with nasal symptom severity, peaking 48–72 h after infection [26].

The SARS-CoV-2 pandemic has underscored the importance of a sophisticated understanding of host‒pathogen interactions to identify factors associated with severity and potential host-directed therapies [31–33]. Transcriptomic approaches aimed at identifying host factors associated with severity in HRV cohorts with severe outcomes are needed and may reveal potential targets for novel host-modulating therapies [34–36]. Furthermore, human experimental models will be key to further understanding the immunology of HRV as well as other respiratory viral infections (RVIs) [37, 38]. A recent review highlighted key pro-inflammatory cytokines involved in HRV infection, including IL-1, IL-4, IL-5, IL-6, IL-25, and IL-33 [39], suggesting their potential as viable targets for cytokine inhibitors to mitigate the inflammatory response induced by HRV.

### HRV transmission

**Fig. 2:**
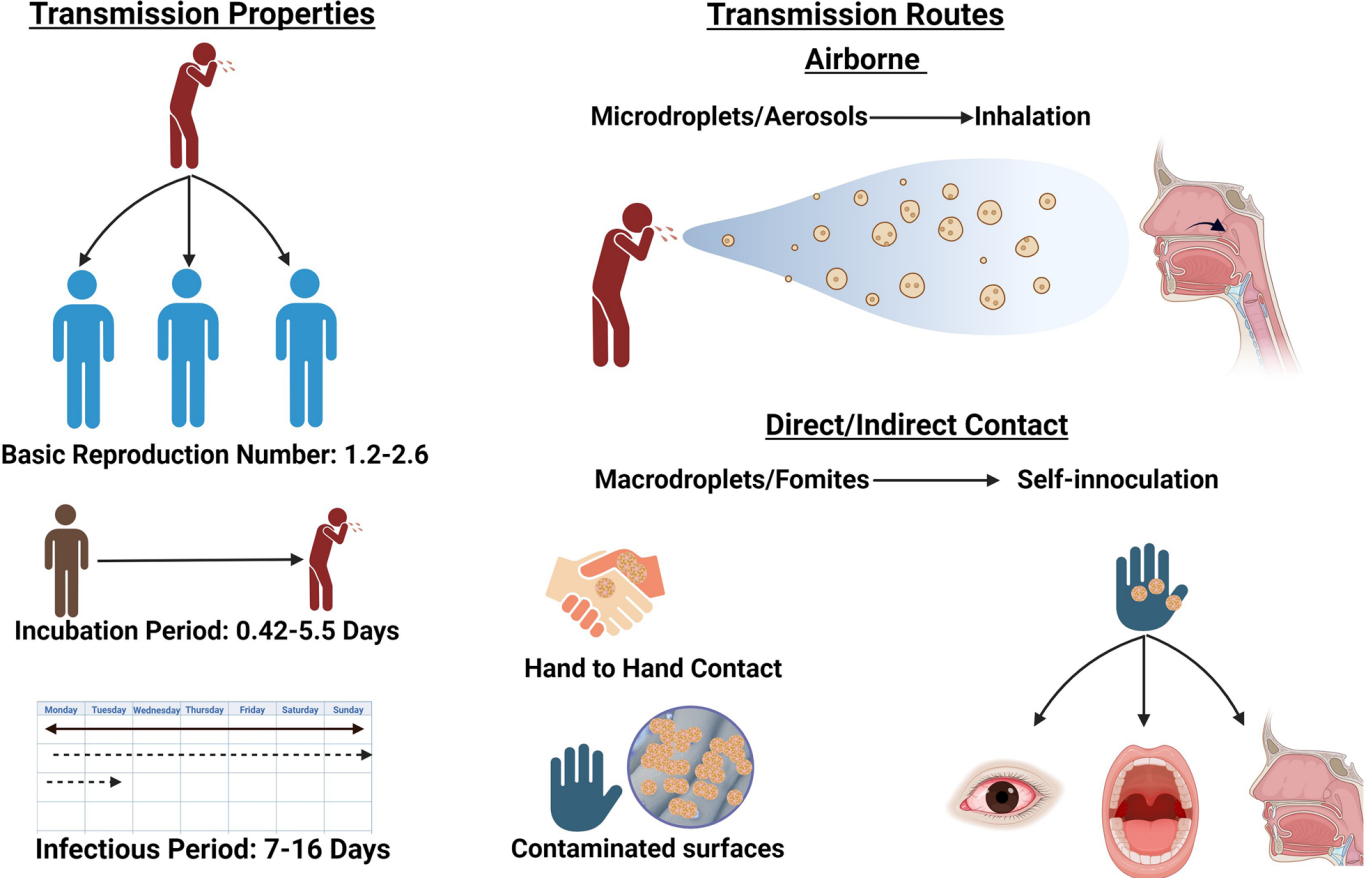
Overview of human rhinovirus (HRV) transmission. HRV is highly infectious and can be effectively transmitted through contact and airborne methods. While contact transmission has been accepted for some time, it now appears that spread via aerosols is likely the dominant method of transmission. HRV can be effectively transmitted during its asymptomatic incubation period

HRV transmission occurs through airborne routes involving droplets and aerosols, as well as via contact through autoinoculation of the nasal or conjunctival mucosa (Fig. 2).

Traditionally, direct or indirect contact with HRV on surfaces (fomites) and subsequent self-inoculation have been considered the primary transmission modes. Gwaltney et al. demonstrated that 11 out of 15 hand-to-hand HRV exposures and 20 out of 28 indirect contact exposures resulted in successful infection in adults [40]. Winther et al. reported that 41% of surfaces in the homes of HRV-infected adults were contaminated with viral RNA, with fresh mucus (< 24 h old) being more likely to facilitate transmission [41]. Turner et al. reported that hand treatments with 2% citric acid and 2% malic acid in 62% ethanol were ineffective at preventing HRV transmission [42]. Alcohol-based handwashing alone has been deemed insufficient to destroy HRV, and traditional soap and water have been suggested to reduce HRV transmission [43].

In the 1980s, Dick et al. suggested that, contrary to the popular opinion at the time, the chief method of HRV transmission was via inhalation of suspended HRV aerosols/microdroplets rather than contact-mediated self-inoculation [44]. This view has gained support through research attention during the SARS-CoV-2 pandemic, highlighting the crucial role that airborne transmission plays in the transmission of respiratory viruses [45, 46]. A recent systematic review suggested that indoor airborne transmission of large or small aerosols is likely the dominant method of HRV transmission [47]. While the transfer of HRV via direct/indirect contact is possible, it is unlikely to be the most common transmission route [47]. This is supported by previous observations that HRV infectivity decreases rapidly upon transfer to hands and surfaces [48]. Factors influencing airborne HRV transmission include strain type, viral load, temperature, humidity, ventilation/filtration systems and ultraviolet radiation exposure [46, 49].

Thus, the most effective methods for reducing transmission are likely to include improving indoor ventilation, maintaining appropriate humidity and temperature levels, and increasing outdoor activities [50]. Notably, surgical facemasks appear to be less effective at preventing HRV spread, although they are somewhat effective at reducing the transmission of enveloped respiratory viruses such as influenza and coronaviruses [51]. One study revealed HRV in 8% of healthcare workers despite adherence to mask policies and contact precautions, highlighting the necessity for improvement in infection prevention measures [52]. Hospital-acquired HRV infection is increasingly recognised [53].

Following infection, HRV has an incubation period of 0.42–5.5 days, an infectious period of 7–16 days and a basic reproduction number (R_0_) ranging from 1.2–2.6 [54].

### HRV circulation patterns

HRV is a ubiquitous perennial pathogen that circulates year-round, with a peak prevalence often observed during spring and autumn [55–59]. This seasonal pattern is often demonstrated across studies focused on adults presenting with upper and lower respiratory illness and acute respiratory infection (ARI) [58–61]. Interestingly, the peak incidences of different respiratory viruses typically do not overlap, which might be influenced by the short-term inhibitory effects of initial HRV infections on other viruses, such as influenza, possibly due to enhanced antiviral IFN responses [62–64]. For example, the prevalent circulation of HRV appeared to delay the onset of H1N1 influenza during the most recent influenza pandemic [62, 65–67].

At the species level, HRV-A and HRV-C are in constant circulation throughout the year, whereas HRV-B circulation is less consistent [57, 59]. A single-centre study from 2013 to 2017, in which respiratory samples were collected from adult patients (65.8% inpatients, 34.2% outpatients), revealed that HRV-A was the predominant species and was detected in 60.9% of patients, followed by HRV-C in 26.4% and HRV-B in 12.7%. HRV-A and HRV-C were detected year-round, whereas HRV-B was not detected from June to August across the 4-year period [59].

The diversity of concurrently circulating HRV genotypes is notable. Even within a single geographic location, a wide array of genotypes can be identified in adults [59], although no single genotype typically constitutes more than 20% of the detected strains [57]. This genotype variability is lower in adults than in children aged 0–3 years [57]. The predominance of any single HRV genotype among adults varies, ranging from 3.9% [59] to 21.4% [68]. Despite differences in circulating genotypes, the proportion of HRV species in symptomatic adults is relatively stable, with HRV-A and HRV-C usually being the most common and HRV-B being the least common species [59, 61, 69].

### Impact of the SARS-CoV-2 pandemic on HRV circulation

Despite widespread lockdowns, social distancing measures, and the use of facemasks during the SARS-CoV-2 pandemic, HRV continued to circulate, even maintaining circulation rates comparable to pre-pandemic HRV levels [70–72]. This contrasts sharply with the incidence of enveloped respiratory viruses such as influenza and respiratory syncytial virus (RSV), which significantly decreased during the SARS-CoV-2 pandemic [73].

A systematic review and meta-analysis explored the pooled prevalence of viruses other than SARS-CoV-2 in symptomatic adults and adolescents during the pandemic [74]. This study suggested that HRVs were by far the most prevalent non-SARS-CoV-2 virus, despite being tested for less frequently than influenza, metapneumovirus (MPV), RSV and human coronavirus. During the first half of the pandemic, the prevalence of HRV was 4.69%, which increased to 9.52% during the second half [74].

A Japanese study during the SARS-CoV-2 pandemic revealed that among 191 patients with upper respiratory symptoms, HRV was the most common viral pathogen identified and was detected more frequently than SARS-CoV-2 [75].

Additionally, a retrospective observational study conducted on Reunion Island among adults with severe community-acquired pneumonia (CAP) requiring intensive care from 2016 to 2021 revealed that HRV was the only non-SARS-CoV-2 viral pathogen whose incidence did not decrease following the onset of the SARS-CoV-2 pandemic. This was noted despite a significant overall decrease in the incidence of non-SARS-CoV-2 CAP between the pre-pandemic period and the early pandemic [76].

## Clinical manifestations of HRV infections in adults

### Spectrum of HRV infections

The clinical manifestations of HRV infection in adults are diverse. Unlike influenza, RSV, or SARS-CoV-2, there are no validated patient-reported outcome measures specifically for adults with HRV infection, beyond generic cold scores. The development of further instruments would be invaluable for objectively assessing patient experiences and serving as outcome measures in clinical trials [77–79]. Additionally, chronic respiratory symptoms in patients with respiratory comorbidities can confound the recognition of acute symptoms associated with HRV as well as other RVIs [80].

We review the respiratory manifestations of HRV infection. It should be noted, however, that non-pulmonary sequelae of HRV infection are emerging. For example, HRV infection has been described as a predictor of myocardial infarction admission in adults aged 65–74 years and stroke admission in adults over 75 years [81], further demonstrating the diverse consequences of HRV infection.

### Upper respiratory infections (URIs)

HRV is the predominant pathogen associated with the common cold, arguably the most frequently occurring human ailment. This condition is often self-diagnosed and deeply ingrained in cultural folklore with numerous suggested origins and treatments [82, 83]. Historically, according to Hippocratic humoral theory, the common cold was believed to stem from excessive cooling that disrupts bodily humours, leading to abnormal mucus production [83]. Modern understanding defines it as a clinical syndrome marked by upper respiratory symptoms such as sore throat, rhinorrhoea, sneezing, nasal congestion, headache, and cough. The term “flu-like illness” is used for more prominent systemic manifestations, including fever, myalgia, and fatigue [82]. Typically, these symptoms often present as a mild prodrome, which becomes progressively worse before peaking and gradually resolving [82].

Research in the 1960s found HRVs are responsible for 10–30% of acute upper respiratory infections in adults [84]. Later studies identified HRV much more frequently. Arruda et al. identified a Picornavirus in 82% of adults with self-diagnosed colds [60]. The first symptom noted in these adults was sore throat, and the illness lasted 9.5–11 days on average [60]. Similarly, Makela et al. identified rhinovirus in 52.5% of adults with clinical evidence of rhinorrhoea, nasal congestion, or sore throat [85].

Adults with the common cold often have sinus involvement. In a study of adults with the common cold who underwent computerised tomography (CT) imaging of the sinuses, 87% had radiological evidence of sinusitis [86]. Acute rhinosinusitis presents with nasal congestion, obstruction, posterior rhinorrhoea anosmia and facial pain. Most cases of acute rhinosinusitis resolve within 10 days, but approximately 3 out of every 100 patients persist longer [87]. Risk factors that modulate susceptibility to the common cold include psychological stress, smoking, disrupted sleep, poor nutrition and older age [82, 88–90].

Although the upper respiratory manifestations of HRV infection are usually self-limiting, they are responsible for immense health, economic and societal costs due to the sheer scale of associated medical visits/prescriptions and loss of work/education [91–95]. The frequency of HRV infection compounds this burden, as both children and adults may be infected by HRV multiple times per year [96–98]. The common cold, for which HRV is primarily responsible, was estimated to cost 4 billion dollars per year, including 1.1 billion dollars on antibiotic prescriptions alone in the US alone in 2003 [94].

### Lower respiratory infections (LRIs)

#### Re-evaluating the role of HRV in LRIs

The term lower respiratory infection (LRI) is often used imprecisely; however, LRIs can be classified as acute bronchitis in the absence of radiological infiltrates or pneumonia in the presence of infiltrates. Community-acquired pneumonia (CAP) is associated with a massive healthcare burden. In the USA, 650 per 100,000 people are hospitalised with CAP every year, with an associated 100,000 deaths [99]. Viruses are implicated in 23–44% of CAPs in adults [100–103]. The role of HRV in LRIs is frequently underestimated.

Traditionally, there has been scepticism regarding the viability of HRV infection of the lower respiratory tract. The optimal rhinovirus replication temperature is 33–35 °C [104], and previous dogma assumed that HRV replication within the warmer lower respiratory tract was unlikely. This assumption is incorrect.

First, although parenchymal lung tissue reaches a temperature of approximately 37 °C [105], the temperature of large- and medium-sized airways is 33–35 °C, which is suitable for HRV replication, and the ideal replication temperature varies between rhinovirus types [106]. Furthermore, ex vivo experiments have suggested that HRVs can replicate more efficiently in the human bronchial epithelium than in the human nasal epithelium [107]. In addition, HRV infection in the lower respiratory epithelium and airway fluid in vivo can be readily demonstrated following experimental infection [108–110]. In the context of natural infection, sputum obtained without contamination from tracheal samples often has a greater amount of HRV than does sputum obtained from secretions from the upper respiratory tract [111]. Finally, HRVs can migrate from the upper respiratory tract to the lower respiratory tract [112]. In support of these observations, many studies in clinical settings confirm the commonality of HRV infection in adults with lower respiratory syndromes, which we discuss below.

#### Evidence for the role of HRV in LRIs from clinical studies

HRV is often identified as the sole respiratory pathogen in lower respiratory samples of acutely unwell adults [113–115]. Minosse et al. reported HRV as the most frequently detected virus in hospitalised adults with lower respiratory infections, accounting for 32.9% of lower respiratory samples, often without co-detection of other pathogens [113]. Similarly, Choi et al. found HRV to be the most commonly identified virus (23.6%) among patients with severe pneumonia requiring ICU admission. Notably, the mortalities of patients with bacterial infections, viral infections, and bacterial-viral coinfections were not significantly different, further suggesting the severity of HRV as a standalone pathogen [114].

While many studies designate HRV as the cause of lower respiratory infection via upper respiratory PCR samples [102], there are inherent challenges in directly confirming causation from upper respiratory swabs. There is high concordance between nasopharyngeal swabs and lower respiratory tract samples, such as bronchoalveolar lavage [116]. Despite this, detection of HRV in the upper respiratory tract alone does not in itself prove causation in adults with lower respiratory symptoms. However, the inclusion of asymptomatic controls in numerous studies strengthens the evidence for causality. These controls consistently report low rates of HRV detection compared to individuals with symptomatic lower respiratory infections, reinforcing the association between HRV and lower respiratory symptoms [61, 102, 117, 118] (Table 1). For example, in the Jain et al. study, HRV was detected in 11% of symptomatic immunocompetent adults hospitalised with pneumonia, whereas it was detected in only 1% of matched asymptomatic controls (*p* < 0.001) [102]. Both groups were recruited during the same period and from the same catchment area, ensuring comparability in terms of demographic and geographic factors. This highlights the potential role of HRV as a pathogen in community-acquired pneumonia among hospitalised adults [102].

**Table 1:** Evidence for the role of HRV in adults with symptoms of lower respiratory infection from studies with matched controls

Study	% HRV in symptomatic LRI	% HRV in Matched Asymptomatic controls	Median duration of symptoms	Most Common LRI symptoms	Setting	P value
Zlateva et al. [61]	19%	4%	HRV-A: 21 days (IQR 14–31), HRV-B: 20 days (IQR 13–31), HRV-C: 19 days (IQR 13–32)	Cough 98-99.6% Sputum 40-50% shortness of breath 31-37% Wheezing 20-26%	Adults with LRI in primary care	P < 0.001
Ieven et al. [118]	20.4%	3.5%	Not stated < 28 day duration	Not stated All acute or worsening cough, GP suspected lower respiratory infection	Adults with LRI in primary care	P < 0.0001
Jennings et al. [117]	10% all patients 13% with full respiratory virus panel	2%	7 days (IQR 3-7)	Cough 94%, Sputum 74%	Hospitalised adults with confirmed CAP	P < 0.001
Jain et al. [102]	11% symptomatic patients with matched control 9% all ages % by Age groups 11%18-49 7% 50-64 8% 65-79 9% 80 +	1%	Median duration illness onset to hospital presentation for all CAP 4 days (IQR 2-7) HRV specific duration not stated	Not stated All clinician confirmed pneumonia	Hospitalised immunocompetent adults with confirmed CAP	P < 0.001

This table summarises evidence from studies comparing the prevalence of human rhinovirus (HRV) in adults with symptoms of lower respiratory infection (LRI) to that in asymptomatic controls. These studies consistently demonstrated significantly greater HRV detection in symptomatic LRI patients, supporting its potential role as a causative agent in LRIs. The key findings included the median symptom duration, most commonly reported symptoms, and study-specific patient settings

Abbreviations: *HRV* human rhinovirus, *LRI* lower respiratory infection, *CAP* community-acquired pneumonia, *GP* General practitioner, *IQR* Interquartile range

Metanalytical evidence further substantiates this relationship. Shi et al. conducted a comprehensive meta-analysis of case–control studies of older adults with ARIs and pneumonia. These findings revealed that HRV was significantly more common in adults aged over 65 years with ARIs or pneumonia than in asymptomatic individuals/healthy controls (OR 7.1; 95% CI 3.7–13.6). Moreover, the study demonstrated an HRV-specific attributable fraction among exposed individuals of 86%, supporting a causal role in these conditions [119]. We will now review the impact of HRV in LRIs in community and hospital settings.

### HRV and LRIs in community settings

LRIs in primary care settings are among the most common reasons for seeking medical attention, contributing significantly to healthcare resource use and morbidity even in otherwise healthy working-age adults. These infections result in an average of 3.5 sick days per year, creating a substantial social and economic impact [120, 121]. HRV is one of the primary pathogens causing LRIs in the community, with multiple studies emphasising its significant role in both healthy adults and vulnerable populations, such as the elderly and individuals with chronic diseases (Table 2). HRV is detected in 20–40% of immunocompetent adults with lower respiratory viral infections in primary care [118, 122, 123].

**Table 2:** Overview of HRV in Lower Respiratory Infections (LRI) Within Community and Primary Care Settings

Community/primary care studies highlighting prevalence of HRV LRI to LRI caused by other viruses				
Authors/year	Patient population	HRV % Detection in RVIs and rank	% of Other common RVIs	Key points
Vos et al. [122]	Adults with acute cough/LRI,	39.7% HRV (Most common virus)	Influenza (13.6%), RSV (9.5%)	HRV associated with more severe symptoms particularly severe wheezing vs virus negative LRI
Ieven et al. [118]	Adults with LRI	20.4% HRV (Most common virus)	Influenza (9.9%), Coronavirus (7.4%)	HRV detected 14.2% of adult pneumonia cases in primary care
Falsey et al. [123]	Elderly adults (≥ 65 years) with moderate-to-severe ILI	25.6% HRV (2.^nd^ most common virus)	Influenza A (18.7%), RSV (7.4%)	14.6% of adults over 65 with HRV LRI ultimately required hospitalisation

Community/primary care studies specific HRV LRI				
Authors/year	Patient population	% HRV detected in all LRI	HRV Species data (symptomatic patients)	Common comorbidities
Zlateva et al. [61]	Adults with acute cough/LRI	19%	HRV-A (68%), HRV-B (12%), HRV-C (20%)	Asthma (12%), COPD (7%), Allergic diseases (23%), Cardiac disease (8%), Diabetes (6%)
Zlateva et al. [98]	Adults with acute cough/LRI	18%	HRV-A (51%), HRV-C (7%), HRV-C (42%)	COPD (29%), Asthma (24%), Allergic disease (29%), Cardiovascular disease (8%)

This table summarises studies investigating HRV in adults with LRI in community and primary care settings. Comparative studies outline HRV detection rates, its rank among respiratory viruses (RVIs), and key insights into clinical presentations. HRV-specific studies highlight species prevalence and associated comorbidities in symptomatic adults

Abbreviations: *HRV* human rhinovirus, *LRI* lower respiratory infection, *RVI* respiratory viral infection, *RSV* respiratory syncytial virus, *ILI* influenza-like illness, *COPD* chronic obstructive pulmonary disease

Cough, sputum production and shortness of breath are the most common lower respiratory symptoms in adults who present to primary care with HRV-associated LRIs [61]. One study demonstrated that HRV infection in adults with community LRIs was associated with greater symptom severity than virus-negative LRIs. Severe wheezing was particularly prominent in HRV-positive LRIs, with an odds ratio (OR) of 1.6 (95% CI 1.9–4.4) [122]. The median symptom duration in adults with HRV LRIs in the community setting ranges from 7 to 25 days [61, 98, 122]. Additionally, 14.2% of adult pneumonia patients in primary care tested positive for HRV, whereas 20.4% of all adults with LRIs tested positive for HRV [118].

Large-scale studies examining community HRV LRIs in adults specifically are scarce, but available research has identified several common comorbidities in immunocompetent adults, including allergic diseases, airway diseases, cardiovascular disease, and diabetes [61, 98]. Furthermore, after initial rhinovirus-associated LRIs, adults with asthma and COPD are at risk of subsequent reinfection with different HRV genotypes, which can be associated with higher symptom scores and longer symptom durations.[98].

Elderly patients are vulnerable to HRV-related LRIs in the community. Community HRV outbreaks are common in nursing home/residential care settings [124, 125]. Hicks et al. described two rhinovirus outbreaks in the United States, and half of the cases in these outbreaks were associated with pneumonia [125]. In a multicentre international study of adults > 65 years of age with moderate–severe influenza-like illness (ILI) in the community (severity defined by the authors as having pneumonia, requiring admission, or having an influenza symptom score > 2), Falsey et al. reported that viruses were detected in 57.6% of cases. HRV was detected in 25.6% of the cases, second only to influenza. Importantly, 14.6% of adults over 65 HRV in the community ultimately required hospitalisation [123].

### HRV in hospitalised adults with LRIs

Historically, HRV pneumonia requiring hospitalisation was thought to be associated primarily with profound immunocompromised states [126, 127]. This perception has been challenged by studies highlighting HRV as a significant pathogen associated with ARI and pneumonia in immunocompetent hospitalised adults.

The EPIC study, a multicentre prospective US surveillance study for CAP in immunocompetent adults, systematically investigated the bacterial and viral aetiologies of CAP in 2259 immunocompetent adults. Respiratory viruses emerged as the most commonly identified pathogens and are detected in 23% of cases. Among all identified pathogens, whether bacterial or viral, HRV emerged as the most frequent, identified in 9% of all CAP episodes [102]. These findings underscore the prominent role of HRV in viral pneumonia even in adults without immunosuppression [102].

In support of these findings, a surveillance study conducted in China similarly revealed HRV in 9% of all CAP cases. The detection rates of HRV were comparable to those of bacterial pathogens such as *Mycoplasma pneumoniae*, *Haemophilus influenzae*, and *Klebsiella pneumoniae* and were higher than those of *Streptococcus pneumoniae*, which is traditionally a leading cause of bacterial CAP [128].

#### Prevalence of HRV in hospitalised adults: comparison with other respiratory viruses

Among respiratory viruses, HRV ranks among the most frequently identified in adults hospitalised with ARI and CAP, worldwide across all adult age groups [58, 113–115, 129–135] (Table 3).

**Table 3:** Rhinovirus prevalence and ranking among respiratory viral infections in hospitalised adults across global studies

Authors/year	Location and setting	Patients	% HRV detected as proportion of RVIs	HRV rank among RVIs
Grech et al. [58]	Regional surveillance, Australia	Adults hospitalised with ARI	Overall: 32.9% Age groups: 18–20: 43.8% 20–70: 36.0% > 70: 28.6%	1st across all age groups
Zimmerman et al. [129]	Regional Surveillance, USA	Adults hospitalised with ARI	30.1%	1st
Chong et al. [130]	Single-centre study, Malaysia	Adults hospitalised with ARI	49.1%	1st
Li et al. [131]	Nationwide surveillance, China	Adults hospitalised with ARI	Age groups: 18–59: 14.1% ≥ 60:17.7%	2nd across all age groups (after Influenza)
Fica et al. [132]	Single-centre study, Chile	Adults hospitalised with ARI	23.7%	2nd (after Influenza)
Bahabri et al. [133]	Single-centre study, Saudi Arabia	Adults hospitalised with CAP	20.7%	2nd (after Influenza)
Hung et al. [134]	Single-centre study, Hong Kong	Adults hospitalised with HRV or Influenza	37.4%	2nd (after Influenza)
Minosse et al. [113]	Multicentre study, Italy	Adults hospitalised with pneumonia or ARI	32.9%	1st
Liu et al. [135]	Nationwide surveillance, China	Adults hospitalised with CAP and SCAP	Age groups: 18–59: CAP: 14.32%, SCAP: 20.16% ≥ 60: CAP:15.41%, SCAP: 16.42%	2nd for CAP and SCAP across all age groups (after Influenza)
Piralla et al. [115]	Multicentre study, Italy	Adults in ICU with CAP	14.6%	2nd (after Influenza)
Choi et al. [114]	Single-centre study, South Korea	Adults in ICU with pneumonia	23.6%	1st

This table summarises global studies assessing the prevalence and ranking of human rhinovirus (HRV) among respiratory viral infections (RVIs) in hospitalised adults with respiratory illnesses, including acute respiratory infections (ARI), community-acquired pneumonia (CAP), and severe community-acquired pneumonia (SCAP). Data are stratified by geographic location, study setting, and patient age groups, illustrating HRV's significant role in hospitalisation across diverse populations and settings

Abbreviations: *HRV* human rhinovirus, *RVI* respiratory viral infection, *ARI* acute respiratory infection, *CAP* community-acquired pneumonia, *SCAP* severe community-acquired pneumonia, *ICU* intensive care unit

A nationwide surveillance study conducted in China between 2009 and 2019 identified HRV as the second most commonly detected respiratory virus in hospitalised adults with ARI, surpassed only by influenza. HRV was detected in 14.1% of those aged 18–60 years and 17.7% of those over 60 years, underscoring the substantial burden of HRV in both younger and older hospitalised adults [131].

In a population-based surveillance study in Pennsylvania, HRV emerged as the most frequently detected viral pathogen in hospitalised adults with ARI from 2015 to 2019, accounting for 30.1% of all viral infections. The annual hospitalisation burden of HRV ranged from 137 to 174 per 100,000 adults, with HRV having the highest virus-specific population burden in adults aged 18–64 years, surpassing influenza, highlighting its impact on working-age populations [129]. This was corroborated by findings from Malaysia, where a recent study detected respiratory viruses in 57% of adults hospitalised with ARI, with HRV contributing to 49% of these cases (Chong et al. 2022).

Similarly, Grech et al. demonstrated that picornavirus was the most prevalent respiratory virus detected in all age groups of adults with respiratory symptoms in a multicentre regional surveillance study from 2014 to 2019. The greatest detection was in adults aged 20–70 years, and picornaviruses accounted for 32.6% of the viruses identified in general medical wards and 40.2% in intensive care units (ICUs) [58]. While the multiplex PCR method used was unable to distinguish between HRV and other *Picornaviridae*, given the scarcity of other Picornaviridae in this age group and clinical context, the vast majority were likely HRV.

#### HRV in ICU settings

The contribution of HRV to CAP extends to more severe cases, including severe CAP (SCAP), which requires ICU admission. A 12-year national surveillance study in China revealed HRV to be the second most common adult viral pathogen in both CAP and SCAP. Among adults aged < 60 years with identifiable respiratory viruses, HRV was detected in 14.32% of non-SCAP cases and 20.16% of SCAP cases. In older adults (≥ 60 years), HRV was present in 15.41% of non-SCAP cases and 16.42% of SCAP cases [135].

Observational studies further highlight the association of HRVs with severe outcomes. Choi et al. identified HRV as the most commonly detected virus in adults hospitalised with SCAP requiring ICU admission, which was detected in 23.6% of cases [114]. Similarly, a European multicentre observational study revealed HRV as the second most common viral pathogen in ICU patients, which was found in 14.6% of cases, second only to influenza [115].

#### HRV and acute respiratory distress syndrome (ARDS)

HRV infection has also been associated with acute respiratory distress syndrome (ARDS) in adults [136–142]. Notably, HRV infection-induced ARDS has been described in immunosuppressed [143] and immunocompetent [136] adults, even in the absence of identifiable bacterial coinfection [137]. These findings highlight the potential of HRV to cause severe respiratory compromise regardless of the host immune status.

### Clinical characteristics and outcomes of HRV infection in hospitalised adults

Few studies have robustly characterised the clinical manifestations and outcomes of HRV infection in hospitalised adults specifically (Table 4). Existing research highlights significant heterogeneity in study populations [53, 59, 130, 132–134, 144]. This variability underscores the importance of cautious interpretation and the need for dedicated large-scale cohort studies to assess the impact of HRV infection comprehensively in this population.

**Table 4:** Characteristics, complications, and mortality in hospitalised adults with human rhinovirus infection

Author/year	Number of patients	Age	Symptoms (% Reported)	Symptom onset prior to admission	Common comorbidities	Length of stay (LOS)	Inpatient complications	Mortality %
Hung et al. [134]	728	Mean 71.6 (SD 20.2)	Fever (42.2%), cough (57%), sputum (53%), rhinorrhoea (6.6%)	Mean 2.8 days (SD 4.8)	Chronic lung disease (22.9%), diabetes (26.4%), cardiovascular disease (21.6%)	Mean 8.7 days (SD 13)	3.7% mechanical ventilation	9.6% (30-day), 14.2% (90-day), 17.2% (1-year)
Fica et al. [132]	32	Mean 79.5 (Range 49–95)	Cough (100%), sputum (81.3%), fever (43.8%), confusion (28.1%), rhinorrhoea (21.9%)	Mean 3 days (Range 1–21)	Chronic lung disease (53.1%), heart disease (34.4%), cerebrovascular disease (31.3%), diabetes (31.3%)	Mean 11.8 days (Range 2–49)	31.2% (28.1% intermediate care, 3.1% ICU)	12.5% inpatient mortality
Boon et al. [144]	550	Median 69 (IQR: 58–78)	Not reported	Not stated	Chronic pulmonary disease (61%), cardiovascular disease (23%), malignancies (26%), renal disease (12%), diabetes (4%)	Not specified	Not specified	8% (30-day)
Chong et al. [130]	240	Mean 62.2 (SD 17.1)	Runny nose (25.4%), sore throat (27.1%), sputum (85%)	Not stated	Hypertension (57.1%), chronic lung disease (50%), asthma (37.1%), diabetes (43.4%), cardiovascular disease (20%)	Not specified	20% mechanical ventilation	5% inpatient mortality
Bahabri et al. [133]	106	Median 71.5 (IQR 44.0–84.0)	Shortness of breath (90.6%), productive cough (62.2%), fever (38.7%)	Not specified	Hypertension (59%), diabetes (50%), chronic respiratory disease (44.3%), heart failure (34.9%)	Median 5 days (IQR 3–8); ICU: Median 10 days (IQR 5–17)	15.1% ICU admission	9.4% (overall), 37.5% (ICU patients)
Golke et al. [59]	187	Mean 54.8 (SD 16.1)	Fever (22.6%)	Not stated	Cardiovascular disease (48.2%), malignancy (41.9%), immunosuppression (38.4%), chronic kidney failure (23.2%)	Median 9 days (Range 1–130)	8.8% ICU admission; 10.6% mechanical ventilation	Not stated
Choi et al. [53]	165	Median 63 (Range 17–91)	Fever (54.3%), cough (76.8%), sputum (72.0%), rhinorrhoea (18.9%)	Median 4 days (IQR 2–7)	Solid tumour (28.5%), diabetes (26.1%), haematologic malignancy (19.4%), COPD (14.5%)	Not reported	Not reported	13.9% (in-hospital); 10.9% (28-day), 18.8% (60-day)

This table provides a comprehensive overview of clinical characteristics, symptom prevalence, comorbidities, inpatient complications, and outcomes in adults hospitalised with human rhinovirus (HRV) infection. The variability in patient demographics, symptom profiles, and mortality rates across studies underscores the complexity of managing HRV-related hospitalisations. These data highlight the need for targeted strategies and population-specific approaches to optimise clinical outcomes

Abbreviations: *HRV* human rhinovirus, *ICU* intensive care unit, *LOS* length of stay, *COPD* chronic obstructive pulmonary disease, *IQR* interquartile range, *SD* standard deviation, *CAP* community-acquired pneumonia

#### Presenting symptoms

Cough, sputum production, and breathlessness are consistently reported as prominent symptoms in adults hospitalised with HRV infection. In contrast, rhinorrhoea and fever are reported less frequently. These findings indicate that reliance on fever or rhinorrhoea as diagnostic triggers, such as in the influenza-like illness (ILI) definition, may lead to underdiagnosis of HRV. Clinicians should maintain a high index of suspicion for HRV even in the absence of these symptoms [53, 133, 134]. Interestingly, the presence of rhinorrhoea was associated with a lower risk of 1-year mortality, as shown by Hung et al. (2017), suggesting that upper respiratory involvement may be representative of an earlier, less severe disease course [134].

Atypical presentations, such as confusion can be a presenting feature, particularly in older adults. For example, confusion was observed in 28.1% of older adults in one study [132], underscoring the importance of considering HRV in elderly patients who may not present with classic respiratory symptoms.

Some studies reported a median duration of symptoms prior to hospitalisation ranging from 2.8 to 4 days [53, 134]. However, prolonged symptom durations of up to 21 days were noted in certain cases [132].

#### Radiological and laboratory findings

Radiological findings associated with HRV pneumonia frequently include bilateral pulmonary infiltrates [132, 133].

Laboratory findings point to potential markers of disease severity, with lymphopenia observed more frequently in ICU patients (71.4% vs. 26.3%, p = 0.01) and elevated neutrophil‒lymphocyte ratios associated with critical cases (10.4 vs. 5.2, p = 0.02) [133]. These markers may provide insights into the inflammatory response to HRV but require further validation in larger cohorts [132–134].

#### Comorbidities and multimorbidity

HRV disproportionately affects older adults, with mean or median ages in hospitalised cohorts ranging from 62 to 79.5 years [130, 132, 134]. Frailty, while underreported in many studies, likely contributes to poor outcomes, particularly among those residing in long-term care facilities. HRV infection in hospitalised adults is commonly associated with significant comorbidities. Chronic lung diseases, cardiovascular disease, diabetes mellitus and hypertension are frequently observed in HRV-positive patients [134], and one study reported that 56.3% of adults hospitalised with HRV had two or more comorbidities [132].

Immunosuppression, although not universally observed, is significant in certain cohorts. For example, one study of hospitalised adults with HRV19.4% had haematologic malignancies, and 28.5% with solid tumours. These underlying states of immunosuppression may have contributed to the relatively higher in-hospital mortality observed in this cohort [53].

#### Inpatient complications and length of stay

The burden of inpatient complications in hospitalised adults with HRV is substantial. ICU admission rates vary widely, ranging from 8.8 to 31.2%, reflecting differences in study populations and healthcare settings [59, 132–134].

Length of stay (LOS) is a key indicator of the healthcare burden associated with HRV. Hospitalised adults with HRV frequently experience prolonged length of stay hospitalisation; for example, one study reported that more than 60% of adults with HRV had a length of stay greater than 7 days highlighting the significant burden HRV places on healthcare resources [132]

#### Mortality

While influenza and is well recognised for its morbidity and mortality, the severity of non-influenza viruses is often underestimated. A prospective 3-year cohort study in adults admitted with ILI revealed that *Picornaviridae* was the most common non-influenza virus detected (27%). Non-influenza viruses have similar lengths of stay and in-hospital mortality (5%), to those of adults hospitalised with influenza [145].

Additionally, a recent cohort study demonstrated that despite having a relatively younger cohort, HRV infected adults had 8% 30-day mortality, which was comparable to mortality in RSV and influenza [144]. Another study focusing on elderly hospitalised adults with pneumonia highlighted that compared with influenza, HRV infection was associated with significantly higher 30-day (9.6% vs. 7.1%, *p* = 0.04), 90-day (14.2% vs. 10%, *p* = 0.006), and 1-year (17.2% vs. 11.7%, *p* = 0.004) mortality rates. [134]. Independent risk factors for 1-year mortality in HRV patients included intensive care unit (ICU) admission (OR: 9.56), living in an elderly home (OR: 2.60), oxygen therapy during hospitalisation (OR: 2.62), and low haemoglobin levels at admission (OR: 2.43) [134]

The pneumonia severity index (PSI) and CURB-65, which are commonly used to stratify the risk of respiratory infections, have shown mixed utility in HRV-related cases. For example, CURB-65 scores ≥ 3 were significantly associated with increased in-hospital mortality [132]. However, another study reported that the PSI did not predict ICU admission in HRV-positive patients [133]. Larger cohorts are needed to validate HRV-specific risk stratification tools.

## HRV and chronic lung disease

### HRV and asthma exacerbations

Theodore Minor associated HRV infection with asthma exacerbations in the early 1970s [146]. HRV has also been implicated in the development of asthma as well as exacerbations [147].

Asthma exacerbations are characterised by acute worsening of symptoms and a decline in pulmonary function and frequently result in emergency department admission, hospitalisation and death [148]. In the United States, 43% of adults experienced at least one exacerbation annually in 2018, despite optimised asthma therapies [149]. Globally, respiratory viruses are responsible for half of all asthma exacerbations in adults, with HRV being the most common viral pathogen [150–153].

Recent meta-analyses have confirmed the significant role of HRVs, with findings showing that HRV was detected in 20–46% of asthma exacerbations in adults [149, 150]. HRV is consistently identified as among the most common causes of asthma exacerbation in adults in both outpatient [151, 153–155] and inpatient settings [151–154, 156]. While patients with and without asthma have a similar frequency of HRV upper respiratory infections, patients with asthma have more frequent and severe lower respiratory symptoms following HRV infection [157].

Recent studies have elucidated age-dependent immunological differences in HRV-specific antibody responses and their links to asthma risk and severity. Recent findings have demonstrated age-dependent differences in the associations between rhinovirus-specific IgG levels and asthma. In children, higher IgG levels specific to HRV-A and HRV-C are linked to increased asthma risk and severity, likely reflecting recurrent infections and heightened immune responses. Conversely, in adults, lower HRV-A, HRV-B, and HRV-C IgG levels are associated with asthma, potentially indicating reduced viral exposure over time or waning immune responses. These findings suggest that immune pathways driving asthma risk evolve across the lifespan, with high HRV-specific IgG levels in children indicative of repeated viral exposure and immune activation, whereas low IgG levels in adults may reflect diminished antigenic stimulation or extended immunity to HRV [158].

HRV infection in asthmatic patients propagates airway hyperresponsiveness and eosinophilic inflammation. A recent meta-analysis demonstrated that adults with asthma exhibit significantly lower levels of IFN-β and IFN-γ than controls following HRV infection [159]. Moreover, elevated levels of IL-4, IL-5, IL-8 and IL-13 correlate with respiratory symptoms in asthmatic patients with HRV infection [159].

Muehling et al. experimentally identified two distinct immunophenotypes among asthmatic adults infected with HRV via analysis of viral load and nasal cytokine profiles [29]. One immunophenotype exhibited increased viral loads along with elevated IFN-α and IL-15. Conversely, patients with other immunophenotypes exhibit a lower viral load but more severe symptoms and a dysregulated immune state with increased IgE and IFN-γ [29]. Additionally, excessive activation of RIG-I in asthmatic patients may impair type I/type III IFN responses, leading to impaired viral clearance and prolonged airway inflammation [160]. HRV infection in asthmatic patients can also induce airway remodelling, highlighting the need for further research to identify potential therapeutic targets aimed at preventing virus-induced remodelling [161]. Understanding these processes could pave the way for precision medicine approaches targeting specific endotypes of HRV-induced asthma exacerbation with host-directed biological therapies.

### HRV and COPD exacerbations

Acute exacerbations of COPD (AECOPD) drives morbidity and mortality in patients with COPD, and viral infections are common triggers [162]. These exacerbations are frequent causes of hospital admission [163].

A landmark prospective study in adults with chronic bronchitis in the 1960s demonstrated that HRV infection could cause acute exacerbations of chronic bronchitis, even in patients without typical upper respiratory symptoms [164]. Seemungal et al. reported that HRV was the most frequently detected viral pathogen and was responsible for 58% of viral exacerbations of COPD [165]. A meta-analysis from 2014 highlighted a prevalence of respiratory viruses in COPD exacerbations of 39.3% (95% CI 36.9–41.6), with a pooled risk ratio of 4.1 (95% CI 2.0–8.5) for AECOPD compared with stable COPD [166]. HRV was identified as a predominant virus with a prevalence of 15% [166]. Many other studies have consistently identified HRV as one of the most common pathogens in AECOPD [152, 163, 165, 167–174].

HRV infections contribute to seasonal peaks in COPD exacerbations, and interestingly, COPD patients colonised with *Haemophilus influenzae* may be particularly vulnerable to HRV-induced exacerbations [171]. Acute viral infection in the context of chronic bacterial colonisation can modulate the host inflammatory response in COPD, involving complex interactions of innate and adaptive responses that drive exacerbations and contribute to other clinical consequences of COPD, such as fixed airflow obstruction, lung remodelling and emphysema [161, 175]. IFN deficiency in COPD, through unclear mechanisms, may increase the susceptibility of patients to a greater viral load and HRV-associated inflammation [162].

### HRV and other lung diseases

The impact of HRV on chronic lung diseases extends beyond asthma and COPD and includes interstitial lung disease (ILD), bronchiectasis, and cystic fibrosis. However, these conditions have been less extensively studied. Evidence indicates that HRV, along with other respiratory viruses, plays a significant role in the exacerbation of these diseases.

Li et al. conducted a retrospective study in patients with viral infections and ILD and revealed that HRV accounted for 9.2% of viral infections in the non-immunocompromised group. Interestingly, non-influenza viral infections are associated with higher 30-day mortality than are influenza infections [176].

In bronchiectasis, respiratory viruses are isolated in approximately a quarter of exacerbations, with HRV contributing to approximately one-fifth of virus-associated exacerbations, ranking second only to influenza in one study [177].

With respect to cystic fibrosis in adults, Flight et al. conducted a prospective study involving 100 adults and reported that respiratory viruses were detected in 30.5% of visits. HRV accounted for 72.5% of the detected viruses, and viral infection was associated with a greater risk of exacerbation (OR 2.26), higher symptom scores, and elevated C-reactive protein (CRP) levels [178].

## HRV infection in profoundly immunosuppressed adults

Profound immunosuppression, such as that observed in patients undergoing hematopoietic cell transplantation (HCT) or lung transplantation, significantly increases susceptibility to severe outcomes from HRV infections. These topics have been reviewed elsewhere, but we provide an overview [179, 180].

### Lung transplant recipients

Lung transplant recipients face unique vulnerabilities to HRV due to the broad immune suppression required to prevent graft rejection. HRV infections in this population are associated with prolonged persistence, lasting up to 12 months, and increased risks of secondary bacterial infections and chronic airway inflammation [181]. HRV is identified as the leading cause of community-acquired RVIs in lung transplant recipients, with a median virologic clearance time of 39.5 days [182].

There is concern that HRV infection contributes to graft dysfunction [181]. HRV has also been implicated in the development of bronchiolitis obliterans syndrome (BOS), a leading cause of chronic lung allograft dysfunction. While evidence for a direct causal link is limited, recurrent RVIs, including HRV, have been associated with small airway injury and progressive airflow obstruction in BOS patients [183]. A meta-analysis of 34 studies revealed no significant association between respiratory viruses and acute rejection, highlighting the need for prospective studies to clarify this relationship [183]. In lung transplant recipients, macrolides such as azithromycin may offer additional benefits. Azithromycin has demonstrated antiviral and anti-inflammatory properties in vitro, including reduced HRV replication and cytokine production [184, 185]. Its clinical use in managing bronchiolitis obliterans syndrome (BOS) has shown improvements in lung function, although whether these effects are due to direct antiviral activity, or the modulation of airway inflammation remains unclear [186].

### Haematopoietic cell transplant recipients

HRV is the most frequently detected RVI in haematopoietic cell transplant (HCT) recipients, accounting for approximately 37% of all infections in this group [187]. In a Geneva cohort of allogeneic HCT recipients, RVIs were detected in 63.5% of patients, with HRV as the predominant pathogen. Co-infections with other viruses are common (26%), reflecting profound immune impairment in this population [187]. HRV infections persist for a median of 26.6 days, with shedding lasting even longer during the first six months posttransplant, a period of incomplete immune reconstitution [187].

Disease severity in HCT recipients is stark: 13–29% of HRV infections progress to LRIs, with 90-day mortality rates reaching 41%, comparable to outcomes observed with RSV, influenza, and parainfluenza virus [188]. These high mortality rates persist even after excluding co-pathogens, underscoring the direct impact of HRV on lung pathology [188]. In a study of myelosuppressed adults with hematologic malignancies, HRV-associated pneumonia resulted in a 32% fatality rate [189].

Several risk factors influence the progression of HRV infections in HCT recipients. Low lymphocyte counts, high-dose corticosteroid use, and positive cytomegalovirus serostatus are significant predictors of severe outcomes [190]. Graft-versus-host disease, a common post-HCT complication, further exacerbates immune dysfunction, increasing the risk of progression to LRTIs and mortality [190]. Persistent HRV shedding, which can last up to 92 days in immunocompromised individuals, complicates infection control and contributes to sustained inflammation and delayed recovery [191]. Pre-transplant HRV infections present additional challenges and can cause concern in whether to proceed to transplant [192]. In the Geneva cohort, 9% of RVIs occurred within 30 days pre-transplant, with 21% progressing to LRIs, and mortality was low. The authors recommended not systematically delaying transplant due to HRV infection, but close monitoring is essential [187].

## Diagnostic challenges in HRV detection

Respiratory viral diagnostics have been extensively reviewed [193, 194]. Reverse transcription polymerase chain reaction (RT‒PCR) is the cornerstone of HRV detection and offers high sensitivity and specificity. Multiplex PCR panels for respiratory viruses are increasingly available to test for a range of respiratory viral pathogens simultaneously.

Historically, the role of viruses in LRIs, including HRV has generally been underappreciated owing to previous reliance on less sensitive technologies. For example, Templeton et al. demonstrated that PCR was more sensitive than traditional culture/serology for the detection of respiratory viruses in a cohort of adults with CAP, where HRV was the most commonly identified viral pathogen [195]. Furthermore, Alimi et al. performed a meta-analysis of 21 European studies of adults with CAP [100]. The proportion of adults with CAP with viral pathogens was 22%, but this percentage increased to 29.0% in studies where PCR was used [100]. As expected, studies after 2010 reported higher proportions of patients with detectable respiratory viruses [100].

Despite significant advancements, challenges persist in detecting and differentiating HRV in clinical settings. These issues are compounded by the underutilisation of HRV testing in clinical practice, the technical limitations of molecular diagnostics, and the lack of standardisation in interpreting results.

### Underutilisation of HRV testing

Although HRV plays a substantial role in acute respiratory infections (ARIs), community-acquired pneumonia (CAP), and exacerbations of chronic lung diseases, routine testing for HRV is not universal in adult clinical settings. Diagnostic workflows often prioritise pathogens such as influenza and respiratory syncytial virus (RSV), leaving HRV underrepresented [193, 196]. While several meta-analyses prior to the SARS-CoV-2 pandemic identified HRV as the first or second most common virus detected in adults with CAP along with influenza, this was despite HRV being tested for less frequently [100, 197, 198]. Thus, the true impact of the HRV is likely underestimated because of the relatively lower frequency of HRV testing [100, 197, 198].

### Diagnostic overlap between HRV and enteroviruses

Most diagnostic panels target the 5′UTR, a region highly conserved across the enterovirus (EV) genus. Thus, most assays are unable to differentiate between HRV and non-HRV EVs. Advanced genotyping of viral capsid proteins (e.g., VP1 or VP4/VP2) allows precise identification but is resource intensive and takes several days, precluding routine implementation [199]. Table 5 summarises the diagnostic performance of several approved and commonly used multiplex assays.

**Table 5:** Comparison of Commercial Multiplex PCR Assays for Detection of Human Rhinoviruses (HRVs) and Enteroviruses (EVs)

Manufacturer and assay	Viruses detected	Sensitivity (%)	Specificity (%)	Notes
BioFire FilmArray Respiratory Panel	HRV/EV	92.7	94.6	Lacks HRV/EV differentiation
GenMark Dx eSensor RVP	HRV only	89.2	96.1	Reports HRV-specific detection Lower sensitivity
Luminex xTAG RVP v1	HRV/EV	100.0	91.3	High sensitivity Lacks HRV/EV differentiation
Luminex xTAG RVP Fast	HRV/EV	95.6	92.5	Faster processing lacks HRV/EV differentiation

This table compares commercially available multiplex PCR assays for detecting human rhinoviruses (HRVs) and enteroviruses (EVs), highlighting differences in sensitivity, specificity, and assay characteristics. Notably, assays differ in their ability to differentiate between HRVs and EVs, with some lacking differentiation capabilities. Data are derived from manufacturer product specifications.

Abbreviations: *HRV* human rhinovirus, *EV* enterovirus, *PCR* polymerase chain reaction, *RVP* respiratory virus panel

Despite the diagnostic overlap, non-HRV EVs are infrequently detected in adults presenting with respiratory syndromes. For example, a surveillance study of respiratory syndromes revealed that only 6.45% of detected Picornaviridae species were non-HRV EVs [200]. Similarly, in a study involving 1500 respiratory samples collected over one year, HRVs accounted for 27.0% of the detections. Non-HRV EVs accounted for only 2.0% [201] of all EVs. Non-HRV EVs predominantly affect children under five years of age and are linked to diverse conditions, including meningitis, encephalitis, paralysis, neonatal sepsis, myocarditis, and hand-foot-and-mouth disease [200, 202].

Among non-HRV EVs, enterovirus D68 (EV-D68) is particularly notable for causing severe respiratory infections. However, its prevalence is generally low outside of sporadic outbreaks, which predominantly affect children rather than adults [203, 204]. During the 2014 EV-D68 outbreak in the United States, 86.9% of cases occurred in children aged 1–17 years, with a peak prevalence in those aged 5–11 years (44.2%). Adults (≥ 18 years) accounted for only 13.1% of the cases reported during this outbreak [204]. Similarly, a meta-analysis reported that children under 5 years of age accounted for 72.1% of EV-D68 cases, school-aged children (5–17 years) for 20.3%, and adults for just 7.6% [203]. Additionally, other non-HRV EVs, such as Coxsackievirus A10 (CV-A10), which is traditionally associated with hand, foot and mouth disease, have also been linked to respiratory infections, albeit typically presenting with milder symptoms than EV-D68 [202, 205].

For most adult respiratory presentations, the inability to distinguish between HRVs and non-HRV EVs is not clinically significant, as the vast majority of cases are attributable to HRV. However, during outbreaks of EV-D68, precise differentiation becomes essential to guide public health responses and clinical management. Clinicians should remain vigilant for non-HRV EVs and consider further classification in cases presenting with atypical features, such as acute paralysis, which may indicate a non-HRV EV aetiology. Additionally, technologies that can differentiate rapidly between HRV species and genotypes may be helpful and become relevant if future treatments are effective against only specific species or genotypes.

### Asymptomatic detection

The detection of respiratory viruses, including HRV, in the absence of symptoms is a well-documented phenomenon. For example, a longitudinal study of 2685 adults visiting a New York tourist attraction over two years reported respiratory virus PCR positivity in 6.2% of samples, with HRV accounting for 50.6% of the positive cases [206]. Notably, more than half of the positive cases were asymptomatic, irrespective of the symptom definition applied [206]. Asymptomatic detection appears to be less prevalent in adults compared to children [207]. In adults, HRV detection rates in asymptomatic individuals range between 4 and 8%. In contrast, children under four years of age exhibit higher rates, ranging from 12 to 33% [61, 208, 209].

Although the prevalence of HRV-positive PCR in asymptomatic adults is not negligible, this should be considered in the context of its significantly higher prevalence in symptomatic adults. Studies that include matched asymptomatic controls provide critical insight into the pathogenic role of HRV. For example, Zlateva et al. reported that HRV was detected in 19% of adults with acute cough or lower respiratory infection (LRI) compared with only 4% of asymptomatic controls [61]. Additionally, Ieven et al. reported that 20.1% of adults with LRIs compared with 3.5% of asymptomatic controls [118] (Table 1).

Viral loads in asymptomatic patients are generally lower [61, 208]; however, significant overlap exists between the viral loads observed in asymptomatic and symptomatic HRV infections. Furthermore, most platforms provide only qualitative positive/negative results without quantitative insights. Cycle threshold (Ct) values, when available from RT‒PCR tests, vary widely across laboratories [194]. However, symptomatic HRV infections are characterised by elevated expression of genes associated with type I interferon, IL-1, IL-12, and IL-6 [210]. Moreover, gene expression profiles in asymptomatic individuals who test positive for HRV are distinct from those in individuals who test negative for respiratory viruses [210]. Thus, the viral load alone may not reliably distinguish between these states. Instead, gene expression analyses offer a more nuanced understanding but are not currently readily available.

The asymptomatic detection of HRV may result from several processes, including detection during the incubation period—prior to symptom onset, during mild infections where symptoms go unnoticed or are considered negligible, or after symptom resolution when viral shedding persists. Five percent of adults with HRV LRIs in the community had positive HRV PCRs on day 28 [98]. However, genotyping revealed that 65% of the prolonged detections involved infection with a new HRV genotype [98]. Prolonged shedding is more common in severely immunocompromised adults [182]. Furthermore, highly sensitive PCR can detect RNA fragments from non-viable HRV from previous infections, further contributing to asymptomatic detection [194]. Clinicians should carefully assess the temporal relationship between reported symptoms and test results and use clinical judgment to interpret positive HRV PCR findings in the context of an individual’s history.

### Reliance on upper respiratory samples

The detection of HRV in upper respiratory tract samples, such as nasopharyngeal swabs, does not confirm active lower respiratory tract involvement. However, studies report high concordance between nasopharyngeal swabs and lower respiratory tract samples, such as bronchoalveolar lavage, supporting the utility of nasopharyngeal swabs in most clinical settings [116]. However, the reliance on nasopharyngeal/upper respiratory sample PCR alone may actually underestimate the true incidence of lower respiratory HRV infection [197]. For example, in adults with CAP, a meta-analysis by Burk et al. revealed that the pooled proportion of adults with CAP with a detectable respiratory virus was 24.5% using nasopharyngeal swabs (95% CI 21.5–27.5%). This proportion increased to 44.2% (95% CI 35.1–53.3%) when only studies that obtained lower respiratory samples from more than half of patients were included [197]. Additionally, Hong et al. detected rhinovirus in 16.7% of nasopharyngeal sample patients but 29.3% of bronchoalveolar lavage samples [211].

## Factors modulating the severity of HRV infection

### Viral factors

A higher HRV viral load has been linked to greater symptom severity, although it does not appear to increase the likelihood of hospitalisation [122, 212, 213].

In adults, among rhinovirus species, HRV-A and HRV-C are more likely to cause more severe symptoms. Conversely, HRV-B is associated with asymptomatic infections. Even in symptomatic cases HRV-B infection, may be milder than HRV-A and HRV-C [61]. In a prospective observational study spanning two years, Chen et al. reported that among 62 healthy adults with ILI, 72.6% were infected with HRV-A, 27.7% with HRV-B, and 9.7% with HRV-C. Compared with HRV-B infections, HRV-A and HRV-C infections were associated with numerically greater upper respiratory, lower respiratory, and systemic symptoms and overall symptom severity scores. HRV-A causes significantly more severe upper respiratory symptoms than does HRV-B infection [214].

Furthermore, a multicentre prospective observational study conducted over four years indicated that 26% of participants tested positive for HRV, with 67.1% being adults. HRV-B infections were significantly less likely to result in hospitalisation (*p* < 0.001) [212]. This finding aligns with in vitro findings showing that, compared with HRV-A and HRV-C, HRV-B replicates more slowly and induces a more attenuated cytokine response [215]. However, in adults hospitalised with HRV there was no significant difference in the length of stay or ICU admission between HRV species [59].

Although one study revealed that HRV-C is more common in adults with asthma and COPD [216], other research has not found convincing evidence that specific HRV species are associated with preexisting respiratory conditions such as asthma or COPD [59, 69].

### Host factors

Hospitalised adults often present with multiple comorbidities, including prevalent conditions such as diabetes, hypertension, and cardiorespiratory diseases [59, 132, 133]. Elderly individuals, immunocompromised patients, and those with multiple comorbidities are particularly at risk for severe HRV infections [124, 132, 133, 217]. Chronic respiratory disease and male sex in particular may be associated with more severe infections requiring intensive care admission [130]. Reduced functional status, such as being bedbound, is identified as a risk factor for hospitalisation due to HRV infection [133]. Current tobacco smoking has also been implicated as a potential risk factor among adults attending the emergency department with HRV infection [218].

Elucidating how the diverse host factors associated with multimorbidity drive impaired outcomes in patients with rhinovirus infection is crucial [219]. Elderly patients may present atypically with attenuated symptoms, potentially due to altered host–pathogen interactions associated with immunosenescence [219]. Immunosenescence involves diminished B-cell production, dysregulated T-cell immunity, thymic involution, reduced naive T cells and a greater proportion of terminally differentiated, functionally impaired, exhausted T cells, thus impacting the response to RVIs [219, 220]. IFNs play a crucial role in antiviral defence and given the diversity of HRV strains, HRVs can largely escape the protection normally provided by the adaptive immune response. Hence, a competent innate immune response is essential. Defective IFN expression has been demonstrated in both asthma patients and COPD patients, which may provide insight into the increased susceptibility of these patients to respiratory viral infection [221–223]. Both immunodeficient and dysregulated immune responses may drive disease severity [224].

### Viral and bacterial coinfection

Viral co-infection has been reported to be common in children; however, in adults, viral coinfection is less common [225–228]. Viral co-infection in adults has been reported to be approximately 1–5% [225, 226, 229–232].

Reported bacterial co-infections vary; one study reported a low prevalence of bacterial coinfections (6.6%) and superinfections (3.8%) [133]. Golke et al. reported that in a hospital centre in Germany, 21% of HRV infections were associated with coinfection. Bacterial co-infection was by far the most common coinfection (71% of all co-infections) [59]. *Staphylococcus aureus, Pseudomonas aeruginosa*, *Haemophilus influenzae*, *Klebsiella pneumoniae,* and *Escherichia coli* were the detected bacterial co-pathogens This study revealed associations between viral-bacterial co-infection and the development of lower respiratory infections. and pneumonia, prolonged length of stay and admission to the ICU [59]. A positive bacterial blood culture has been associated with a greater risk of critical illness in adults with HRV infection [130].

Bidirectional interactions between bacteria and viruses may occur, whereby colonisation of the respiratory epithelium by bacteria may increase susceptibility to HRV infection. Conversely, HRV infection can disrupt epithelial integrity, potentially predisposing individuals to secondary bacterial infections [233]. A recent experimental study in 581 healthy adults demonstrated that HRV infection significantly increased the risk of secondary pneumococcal colonisation following intranasal bacterial challenge [234]. HRV infection is strongly associated with increased bacterial density and enhanced pneumococcal shedding via the nasal route, suggesting that infected adults may act as reservoirs for bacterial transmission [234].

Notably, while HRV-associated CAP requiring ICU admission has been described without identifiable bacterial coinfection, high rates of antibiotic prescription may lead to negative culture results, masking the influence of bacterial pathogens [133].

Interestingly, a recent systematic review revealed that, owing to the limited number of studies, there is no evidence that pneumococcal conjugate vaccines provide protection against severe outcomes in adults with rhinovirus infection, which contrasts with some evidence for protection against influenza and coronavirus infections [235].

Concern regarding bacterial coinfection and secondary bacterial infection in the presence of HRV detection, particularly in the community, is a leading cause of antibiotic prescription, despite their ineffectiveness in most cases. Unnecessary antibiotic prescriptions by clinicians are a key driver of the growth of antimicrobial-resistant organisms [236, 237]. Clinicians need valid, cost-effective tools to distinguish between bacterial and viral infections.

Identifying bacterial coinfection remains challenging, highlighting the need for novel biomarkers to guide appropriate antibiotic use. FebriDx is a rapid point-of-care immunoassay that measures myxovirus resistance protein A (MxA) and C-reactive protein (CRP) from a finger-prick test [238]. It has shown promising sensitivities of 92.2% for bacterial infection and 70.3% for viral infection, with specificities of 88.4% and 88.0%, respectively [238]. Transcriptomic profiling may also hold promise for identifying bacterial co-infection, particularly in acutely unwell patients where obtaining invasive lower respiratory samples for culture/PCR can be challenging [239–242].

## HRV therapeutic challenges

As mentioned above, HRV is frequently underrecognized. Rapid detection of HRV will be a prerequisite for timely delivery of HRV therapies in acutely unwell adults, although specific therapies are currently lacking [100, 197, 198].

Current treatments for HRV infection are supportive. Analgesics/antipyretics such as paracetamol, ibuprofen, and aspirin are used to alleviate symptoms such as fever, headache, muscle aches, and sore throat. Nasal congestion, although temporarily relieved with sympathomimetics such as xylometazoline and pseudoephedrine, often recurs upon cessation of treatment [82].

Several promising treatments are currently in development, including capsid binders, viral enzyme inhibitors, and host-targeted antivirals [243, 244].

Capsid binders act by preventing viral uncoating post-entry into host cells, binding to VP-1 and thereby inhibiting the release of HRV RNA into the cytoplasm [245]. Pleconaril an oral capsid-binding antiviral has shown efficacy in reducing viral RNA levels and culture positivity, as well as in shortening illness duration by approximately one day. However, its effectiveness is influenced by viral susceptibility, and up to 10.7% of post-treatment virus isolates may exhibit partial or total resistance. Pleconaril is also associated with notable side effects, including interference with contraceptive efficacy and moderate gastrointestinal upset [245, 246]. Pirodavir, another capsid binder administered intranasally, has been shown to reduce viral shedding but has no significant clinical benefit on symptom duration [247]. A key limitation of capsid binders is their inability to effectively target HRV-C species [248] and concerns regarding the induction of antiviral-resistant HRVs [249]. Viral enzyme inhibitors, such as rupintrivir, which inhibits the picornavirus 3C protease, show potent activity against HRV, although their clinical efficacy remains uncertain [250]. Additionally, gemcitabine, which was originally used in cancer therapy, exhibits viral enzyme inhibitor properties by inhibiting viral polymerase activity in enteroviruses when it is administered at lower doses [251].

Host-targeted antivirals represent another approach, with compounds such as 25/27-hydroxycholesterol reducing phosphatidylinositol 4-phosphate on the endoplasmic reticulum, thereby preventing the recruitment of viral RNA polymerase [252]. These drugs show potential for broad-spectrum antiviral activity [253, 254]. Other host-targeted antivirals include phosphatidylinositol 4-kinase IIIB (PI4KB) inhibitors, which also have potentially broad-spectrum antiviral activity. However, there have been concerns regarding their toxicity to the host [255, 256].

The use of IFNs to enhance host anti-viral defence has also been explored. Nebulised IFN-β has been studied for potential use in viral exacerbations of asthma and SARS-CoV-2 infection, and although not meeting primary endpoints, there is some evidence of a signal suggesting prevention of progression to more severe disease. However, further studies are required to investigate these findings [257, 258].

## Vaccine Challenges

Efforts to develop effective HRV vaccines have been ongoing since the 1960s, when it was established that deliberate inoculation could induce protective antibodies against specific HRV strains [259]. Subsequent studies demonstrated that intramuscular administration of inactivated HRV could lead to the production of antibodies capable of protecting against subsequent illness [260–262].

However, developing vaccines with prolonged and broad immunity has proven challenging owing to the vast number of co-circulating HRV types and their associated antigenic diversity [263–265]. Promisingly, a polyvalent vaccine targeting 50 HRV strains has been developed and tested in macaques [266]. Practical strategies include regular surveillance of HRV in acutely ill populations and focusing vaccine efforts on the most prevalent and pathogenic HRV types. Additionally, vaccines that target common epitopes shared among HRV strains to induce cross-strain immune responses are in development [267]. These vaccines could mitigate the challenges posed by the antigenic diversity of HRVs. Vaccines are particularly crucial for at-risk groups, such as patients with COPD, with the aim of reducing the frequency of virus-induced exacerbations [268].

## Conclusion

**Fig. 3:**
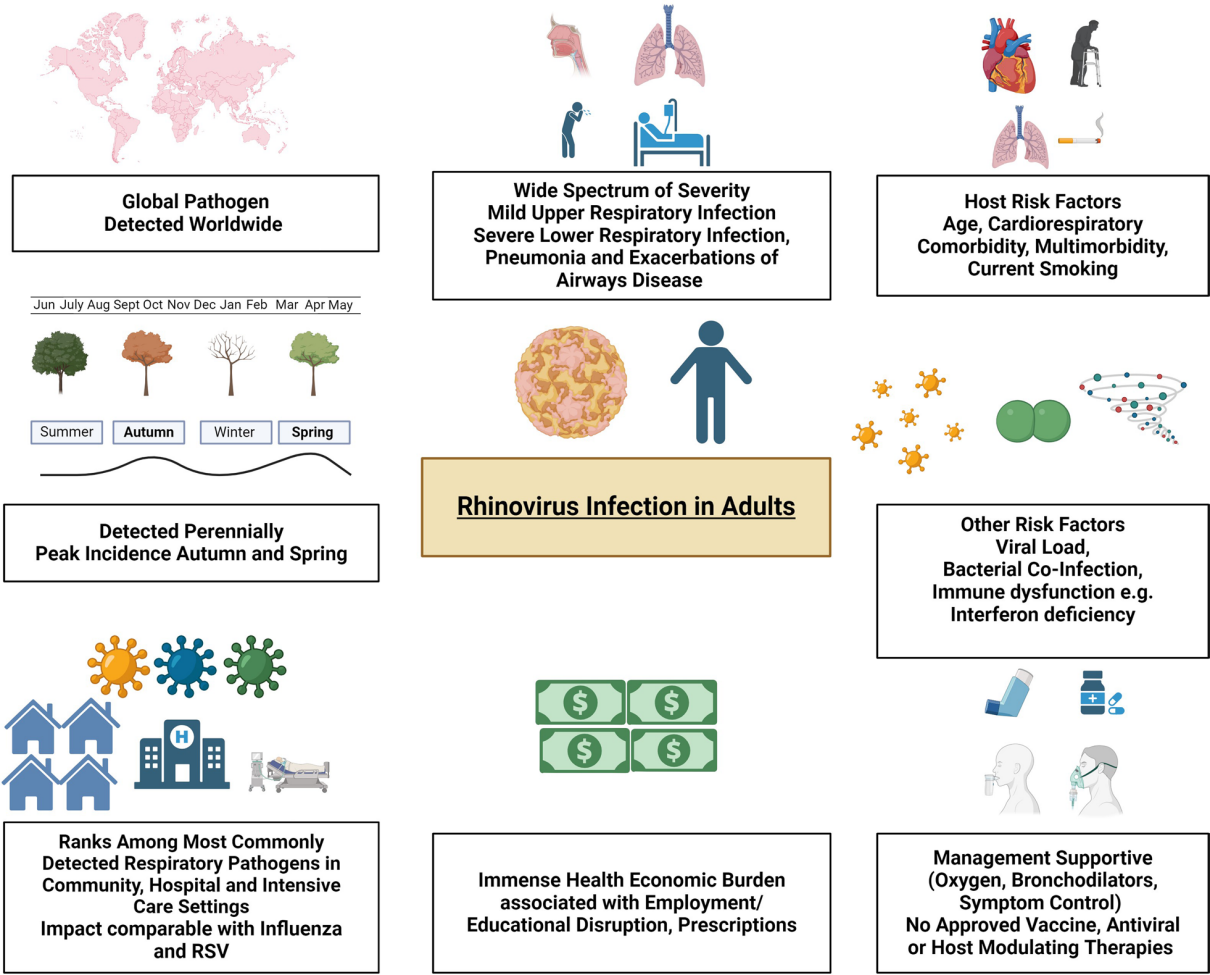
Impact of rhinovirus infection in adults. HRV is a major global pathogen and is detected throughout the year. Upper respiratory manifestations, including the common cold, are the most common manifestation and while self-limiting are associated with a considerable societal cost. It is now increasingly apparent that HRV is a driver of severe lower respiratory manifestations in adults, including exacerbations of airway disease and pneumonia, on a scale comparable to that of other respiratory viruses. The severity of infection is determined by the complex interaction of host factors such as age, multimorbidity and immune dysfunction alongside other factors such as viral characteristics and the presence of bacterial co-infection. There is an urgent need for novel treatment options, as options currently available are merely supportive in nature

HRV is not merely the ubiquitous agent of the common cold; it is a predominant pathogen in adults presenting with ARI in diverse healthcare settings—from primary care to intensive care units (Fig. 3). This pathogen significantly influences morbidity, mortality, and hospital stay duration across a wide spectrum of adult populations. Elderly, immunocompromised, and individuals with cardiorespiratory comorbidities are particularly vulnerable to severe outcomes. HRV transmission through aerosolisation contributes to its persistence and prevalence throughout the year. The dogma that HRV infection is confined to mild upper respiratory infections should be challenged, as HRV is frequently implicated in severe lower respiratory conditions, even in immunocompetent adults, including pneumonia and exacerbations of asthma and COPD.

Given its extensive impact, it is important to improve our understanding of HRV epidemiology, comparable to SARS-CoV-2, to enhance our understanding of the strain-specific severity of infections. More prospective observational studies and continuous surveillance are needed, particularly among community care homes and among hospitalised adults [269]. This approach would not only facilitate the identification of high-risk HRV strains to guide vaccine development but also aid in precisely determining factors that influence the severity of infections and host‒pathogen interactions.

Such data are essential for developing effective intervention strategies, including targeted antiviral therapies and vaccines. Given the scale of HRV infection, the use of digital tools to assist with the design of future care pathways and clinical trials may be valuable [270]. Moreover, improving methods for detecting bacterial coinfection is critical, as they could guide appropriate antibiotic prescriptions and potentially reduce the burden of severe infections.

By elevating HRV to a status comparable to that of other major viral pathogens, such as influenza, in research and public health priorities, we can better mobilise resources and direct efforts towards mitigating its substantial impact on public health. This comprehensive approach is essential for developing effective preventive and therapeutic measures that could lead to significant reductions in severe ARI cases associated with HRV.

## Data Availability

No datasets were generated or analysed during the current study.

## Abbreviations

AECOPD: Acute exacerbations of COPD
ARDS: Acute respiratory distress syndrome
ARI: Acute respiratory infection
CAP: Community acquired pneumonia
COPD: Chronic obstructive pulmonary disease
CI: Confidence interval
CRP: C-Reactive protein
CT: Computerized tomography
CHDR-3: Cadherin related family member 3
ENA-78: Epithelia neutrophil activating peptide 78
MxA: Myxovirus resistance protein A
H1N1: Haemagglutinin 1 neuraminidase 1
HRV: Human rhinovirus
HCT: Hematopoietic cell transplant
ICU: Intensive care unit
IL: Interleukin
ILI: Influenza like illness
IFN: Interferon
IP-10: Interferon gamma inducible protein 10
ICAM-1: Intracellular adhesion molecule 1
LDL-R: Low density lipoprotein receptor
LRI: Lower respiratory infection
MDA-5: Melanoma differentiation associated gene
Non-HRV EV: Non-human rhinovirus enterovirus
OR: Odds ratio
PAMP: Pathogen associated molecular pattern
PI4KB: Phosphatidylinositol 4-Kinase IIIB
PCR: Polymerase chain reaction
PRR: Pattern recognition receptor
PSI: Pneumonia severity index
RANTES: Regulated upon activation, normal T cell expressed and secreted
RIG-1: Retinoic acid inducible gene 1
RNA: Ribonucleic acid
RSV: Respiratory syncytial virus
RT-PCR: Reverse transcription polymerase chain reaction
SARS-CoV-2: Severe acute respiratory syndrome coronavirus 2
TLR: Toll like receptor
URI: Upper respiratory infection
VP: Viral protein
